# MRI-based assessment of the pineal gland in a large population of children aged 0–5 years and comparison with pineoblastoma: part II, the cystic gland

**DOI:** 10.1007/s00234-016-1683-0

**Published:** 2016-04-29

**Authors:** Selma Sirin, Marcus C. de Jong, Paolo Galluzzi, Philippe Maeder, Hervé J. Brisse, Jonas A. Castelijns, Pim de Graaf, Sophia L. Goericke, on behalf of the European Retinoblastoma Imaging Collaboration (ERIC)

**Affiliations:** Department of Diagnostic and Interventional Radiology and Neuroradiology, University Hospital Essen, Essen, Germany; Department of Radiology and Nuclear Medicine, VU University Medical Center, PO Box 7057, 1007MB Amsterdam, The Netherlands; Unit of Diagnostic and Therapeutic Neuroradiology, Department of Neurosciences, Azienda Ospedaliera Universitaria Senese, Siena, Italy; Department of Radiology, University Hospital, Lausanne, Switzerland; Department of Radiology, Institut Curie, Paris, France

**Keywords:** Pineal gland, Pineoblastoma, Retinoblastoma, Pediatric, Gland size

## Abstract

**Introduction:**

Pineal cysts are a common incidental finding on brain MRI with resulting difficulties in differentiation between normal glands and pineal pathologies. The aim of this study was to assess the size and morphology of the cystic pineal gland in children (0–5 years) and compare the findings with published pineoblastoma cases.

**Methods:**

In this retrospective multicenter study, 257 MR examinations (232 children, 0–5 years) were evaluated regarding pineal gland size (width, height, planimetric area, maximal cyst(s) size) and morphology. We performed linear regression analysis with 99 % prediction intervals of gland size versus age for the size parameters. Results were compared with a recent meta-analysis of pineoblastoma by de Jong et al.

**Results:**

Follow-up was available in 25 children showing stable cystic findings in 48 %, cyst size increase in 36 %, and decrease in 16 %. Linear regression analysis gave 99 % upper prediction bounds of 10.8 mm, 10.9 mm, 7.7 mm and 66.9 mm^2^, respectively, for cyst size, width, height, and area. The slopes (size increase per month) of each parameter were 0.030, 0.046, 0.021, and 0.25, respectively. Most of the pineoblastomas showed a size larger than the 99 % upper prediction margin, but with considerable overlap between the groups.

**Conclusion:**

We presented age-adapted normal values for size and morphology of the cystic pineal gland in children aged 0 to 5 years. Analysis of size is helpful in discriminating normal glands from cystic pineal pathologies such as pineoblastoma. We also presented guidelines for the approach of a solid or cystic pineal gland in hereditary retinoblastoma patients.

**Electronic supplementary material:**

The online version of this article (doi:10.1007/s00234-016-1683-0) contains supplementary material, which is available to authorized users.

## Introduction

Pineoblastoma presents in about 3–4 % of children with hereditary retinoblastoma typically within the first 5 years of age [1, 2]; the combination of hereditary retinoblastoma and pineoblastoma is also referred to as trilateral retinoblastoma. The differentiation between cystic variants of pineoblastoma and pineal cysts, which have been reported to appear similar on MRI [3–5], is of high clinical importance, because survival has been reported to be much better in asymptomatic patients with small tumors [1]. Asymptomatic patients showed a 5-year survival of 50 % whereas of patients with symptomatic disease, only 4 % survived [1], emphasizing the importance of early detection. Additionally, it has recently be shown that abnormal growth of the pineal gland might be the most alerting sign for pineoblastoma and that the size of the pineal gland is comparable between retinoblastoma patients without pineoblastoma and age-matched controls [6]. Therefore, normal values of the size of cystic pineal glands in non-retinoblastoma patients in this age group are expected to be helpful in this differentiation.

Several aspects have to be considered in the size evaluation of the pineal gland in children. Due to the high incidence [7, 8], pineal cysts are usually rated as normal variant [9], although they might sometimes be symptomatic requiring treatment [10]. Compared to solid (non-cystic) pineal glands, a higher interindividual variability has been postulated for the size of the pineal gland in the presence of pineal cysts [8]. Additionally, several studies showed that the size of the pineal gland is age-dependent especially in the first years of age [7, 8, 11]. Al-Holou et al. showed that younger age was associated with cyst change or growth [12], which might also result in higher intraindividual and interindividual variability. These aspects reflect the problematic rating of the size of the cystic pineal gland as normal or enlarged that the radiologist and clinicians are faced with especially in young children in the first years of age.

The aims of this retrospective study were (1) to establish normal values for the size of the cystic pineal gland in children 0–5 years in a large patient group, (2) to evaluate the normal morphology of the cystic pineal gland, (3) to assess the development of the cystic pineal gland in those children that received a follow-up, and (4) to compare the results with the results of a large collective of children with pineoblastoma. The solid pineal gland was analyzed in part I of this study. Finally, we present a flowchart for follow-up of pineal glands in retinoblastoma patients.

## Material and methods

The retrospective study was approved by the institutional review boards.

### Patients

This retrospective study included patients from four European neuroimaging or radiology departments of university hospitals in Amsterdam, Essen, Lausanne, and Siena. Inclusion criteria for this retrospective study were (1) the acquisition of sagittal T2-weighted sequences of the pineal gland with a slice thickness of not more than 2 mm in patients without any known or visible pineal pathology and (2) an age 0–5 years at time of the MRI. Exclusion criteria were the diagnosis of retinoblastoma, known endocrinologic or neurological disorders (possibly) affecting or related to the pineal gland, pineal pathologies or distortion of the pineal gland from adjacent pathologies, ongoing radiation therapy or chemotherapy, and relevant MR artifacts at the level of the pineal gland.

Glands with a cystic part were present in a total of 257 examinations of 232 patients (55.8 % of all included patients, *n* = 216 examinations of 191 patients from Essen, *n* = 25 examinations of 25 patients from Siena, *n* = 11 examinations of 11 patients from Amsterdam, and *n* = 5 examinations of 5 patients from Lausanne). These patients are evaluated in this study; the solid pineal glands (184/416 patients, 44.2 %) were assessed separately, see part I of this article. Indications for imaging were not related with pineal gland alterations; MRI was mainly performed in children with developmental retardation, brain malformation, seizures, trauma, prematurity, neonatal asphyxia, infectious disease, hydrocephalus, and pathologies in other parts of the brain. Mean age at the time of the first MR examination was 23.3 months (SD 17.1, range 0–60 months).

### MR imaging

Due to the multicenter setting of this study, the examinations were performed on different 1.5 and 3.0 Tesla MR systems (Magnetom Avanto, Aera, Symphony or Skyra, Siemens Healthcare, Erlangen, Germany) and different T2-weighted sequences were used. We only included MR examinations if the sagittal T2-weighted sequences had a slice thickness of no more than 2 mm to minimize partial volume effects. The slice thickness of the included patients varied between 0.6 and 2 mm.

### MR data analysis

**Fig. 1:**
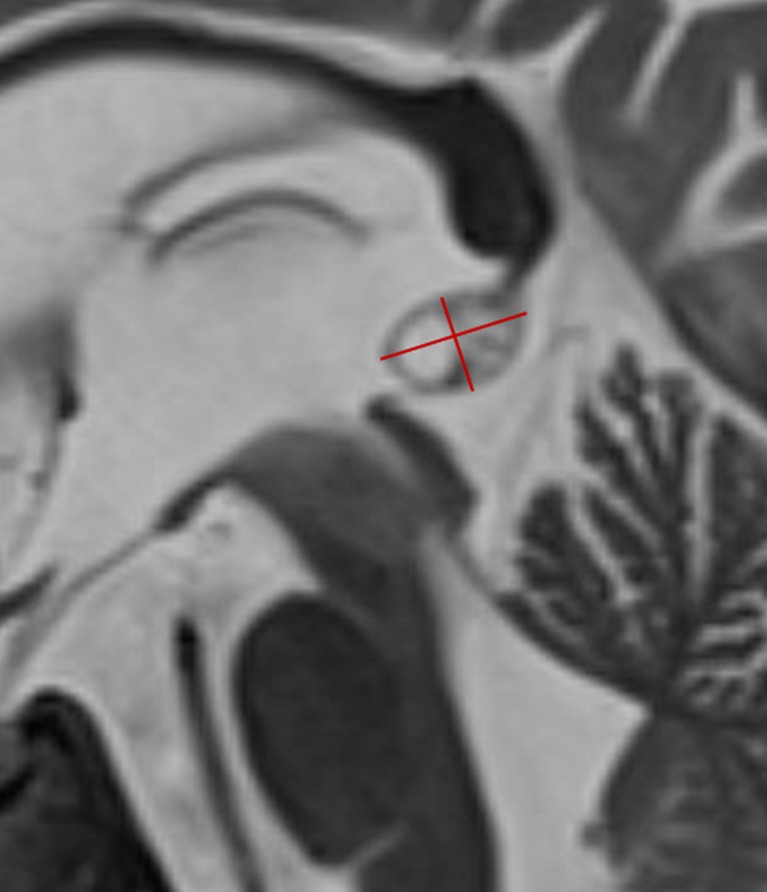
The largest anteroposterior (width) and craniocaudal (height) diameters of the pineal gland were measured as shown in this sagittal T2-weighted image

The datasets were anonymized prior to analysis. All pineal glands were assessed by four senior neuroradiologists (S.L.G., P.d.G., P.G., and P.M.) with 12, 12, 17, and 26 years of experience, retrospectively. The largest anteroposterior (width) and craniocaudal (height) diameters of each pineal gland were reported on the sagittal T2-weighted sequences (as shown in Fig. 1), and the planimetric area (A) was calculated according to the formula A = (width/2) ⨯ (height/2) ⨯ π. Additionally, the maximum diameter of the pineal cyst or the cystic part of the pineal gland (in patients with multicystic pineal gland) was measured. Additionally, the morphology of the pineal gland was classified according to a newly proposed classification system for pineal glands (type 0 to 4) shown in Table 1, which was generated and approved by all involved radiologists in consensus. A suffix was added for type 1 to 4 considering the size of the cyst(s) (a ≤5 mm, b 6–9 mm, and c ≥10 mm).

**Table 1:** Classification system for the (cystic) pineal gland

Type	Definition
0	No cyst
1	Singular cyst
2	Multicystic pineal gland (without enlargement)
3	Multicystic pineal gland (enlargement without shift of the margin)
4	Multicystic pineal gland (enlargement and shift of the margin)

### Statistical analysis and comparison with pineoblastoma

The statistical analysis is similar to part I of this article, except, thanks to a more even distribution of cases across the age interval, created five age categories of 1 year in this part. The clinical usefulness of the age-dependent prediction intervals were compared with pineoblastomas from the meta-analysis by De Jong and colleagues ([1], see part I for more details). The results from parts I and II of this article are combined in a flow chart. During a consensus meeting of the European Retinoblastoma Imaging Collaboration (ERIC), these guidelines were constructed.

## Results

### Size of the cystic pineal gland and the pineal cyst(s)

The measurements of pineal width, height, and cyst size showed ICCs of 0.995 (95 % confidence interval [CI] 0.989–0.998), 0.996 (95 % CI 0.992–0.998), and 0.998 (95 % CI 0.996–0.999), respectively.

**Fig. 2:**
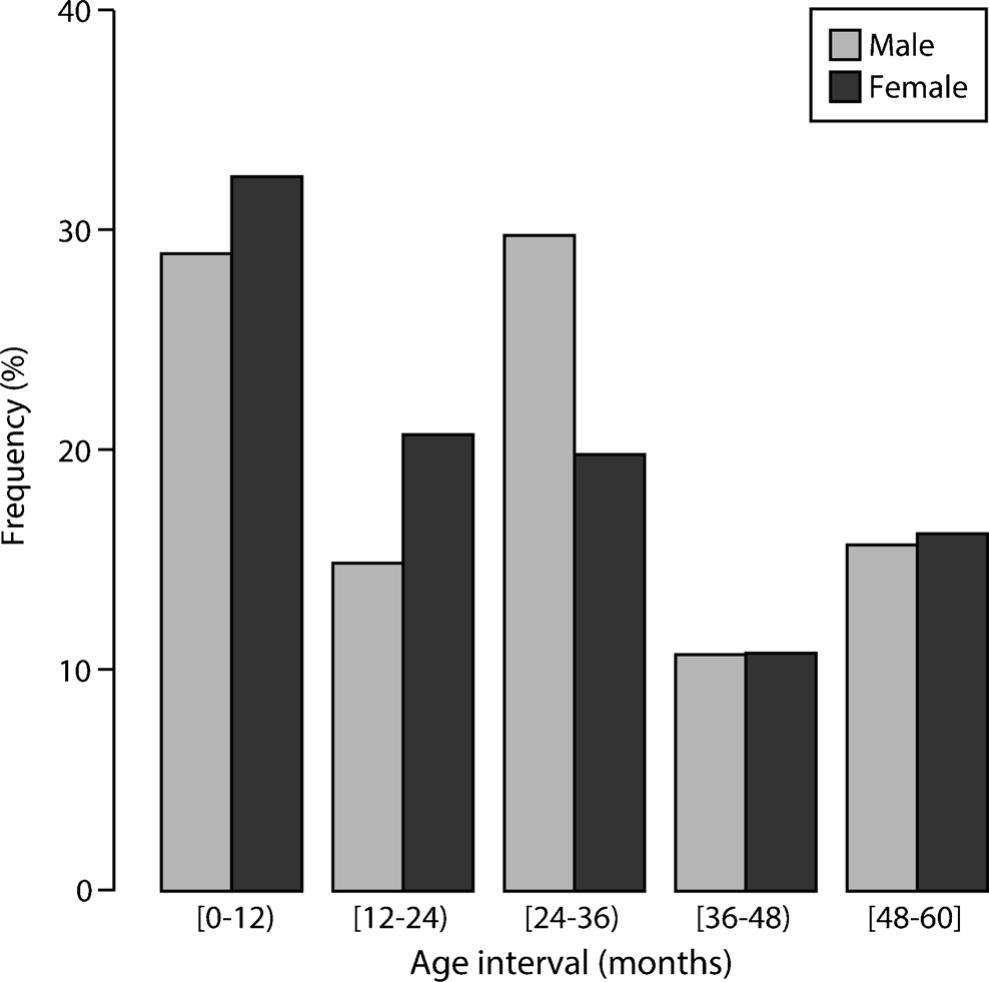
Frequency distribution of cases across the size categories by gender

Figure 2 shows the distribution of male and female cases for each age category (52.2 % male, 47.8 % female). The Kolmogorov-Smirnov test did not reject the null hypothesis that the variable age of female and male subsamples came from the same continuous distribution (*p* = 0.37). The *χ*^2^ test did not show a statistically significant interaction between age and gender; therefore, we assumed that the age distribution did not relate to gender (*p* = 0.53). Levene’s test showed that the homoscedasticity assumption (homogeneity of variance) was met by all gland size variables (area had to be log transformed) across the age intervals by gender (Appendix A). The two-way ANOVA showed that age significantly predicted the size variables (width, height, area, and cyst size), whereas gender did not predict gland size. None of the interaction terms were statistically significant for gland size, but the interaction term age*gender was statistically significant for cyst size (Appendix A). Post hoc analysis suggests that especially in the first year of age, the size parameters increased substantially; all size parameters were significantly lower than in the other age categories (Appendix B).

**Fig. 3:**
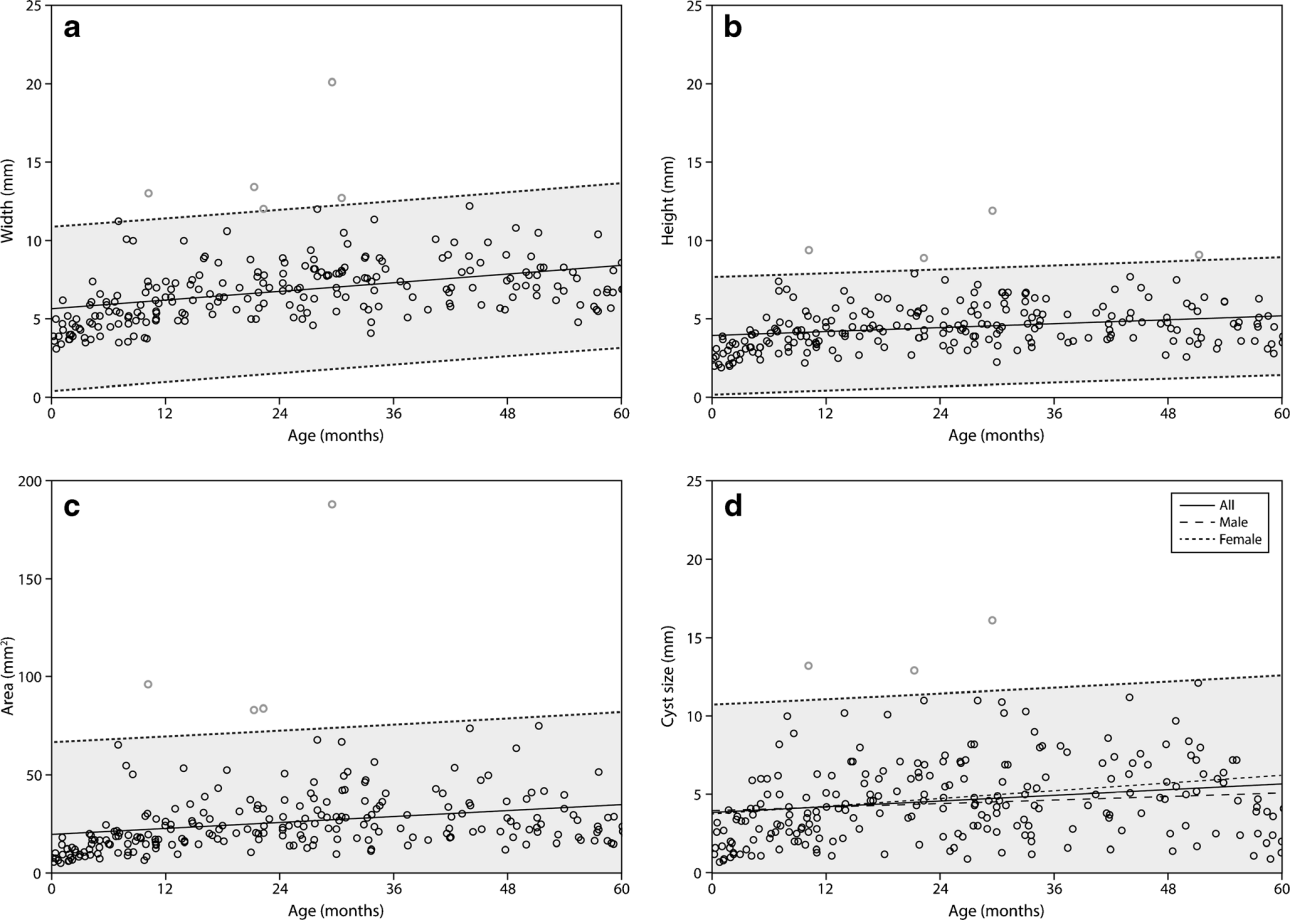
Linear regression line with 99 % prediction intervals of width (**a**), height (**b**), area (**c**), and cyst size (**d**)

Figure 3 shows the linear regression analysis of pineal width, height, area, and cyst size, with each having a 99 % prediction interval. The upper (and lower) 99 % prediction intervals approach linearity and therefore share the slope with the regression line (Table 2). Because the interaction term age*gender was statistically significant for cyst size, we also plotted the separate regression lines for male and female (Fig. 3d); even though the regression lines of male and female patients indeed deviate, the differences are not large and based on visual assessment differentiation by gender is not necessary, and the summary regression line and 99 % prediction interval can be used in our view. The data suggests a rapid size increase in the first year(s) of age and a decrease towards the fourth and fifth year of age, which might be better described by a quadratic function (Appendix C). We therefore also plotted quadratic regression line with 99 % prediction interval for each size variable (Appendix D). Appendix E shows the results from quadratic regression of all size variables. The values of the adjusted *R*^2^ of the regression analysis showed a better fit for all size parameters in quadratic regression compared to linear regression (Table 2 and Appendix E).

**Table 2:** Results of linear regression analysis: cystic pineal gland size versus age

Relationship	Mean intercept (mm)	Upper bound (mm)^a^	Slope (mm/month)	*p* value	Adjusted *R* ^2^
Cyst size^b^ vs. age	3,8	10.8	0.030	0.0025	0.035
Width vs. age	5.7	10.9	0.046	<0.0001	0.137
Height vs. age	4.0	7.7	0.021	0.0001	0.058
Relationship	Mean intercept (mm^2^)	Upper bound (mm^2^)^a^	Slope (mm^2^/month)	*p* value	Adjusted *R* ^2^
Area vs. age	19.6	66.9	0.25	0.0002	0.053

^a^The upper 99 % prediction bound approaches linearity; therefore, the slope of the linear regression line can be used

^b^Maximum diameter of the cyst(s) within the pineal gland

### Morphology of the cystic pineal gland and classification

**Fig. 4:**
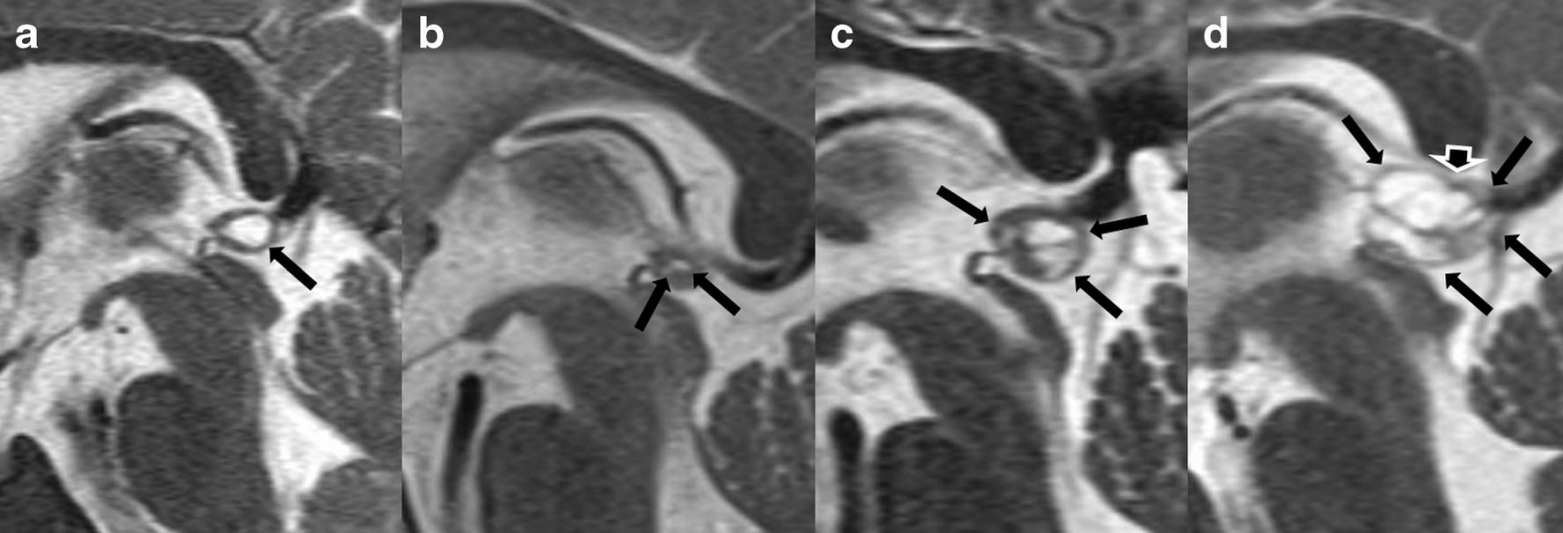
Examples for the cystic pineal gland classification (*arrows*: pineal cysts, *arrowhead*: marked shift of the margin of the pineal gland). **a** Singular pineal cysts rated 1b, **b** multicystic pineal gland without enlargement rated 2a, **c** multicystic pineal gland with enlargement without shift of the margin rated 3b, and **d** large multicystic pineal gland with enlargement and shift of the margin rated 4c

Of the 232 patients, 45.3 % showed a singular cyst (mean size 3.0 mm (SD 2.1, range 0.7–11.2 mm)) and 54.7 % a multicystic pineal gland (mean size of the cystic part 5.9 mm (SD 2.4, range 2.0–16.1 mm)). The classification of the cystic pineal glands according to the classification system is shown in Table 3; examples are shown in Fig. 4. Appendix F shows the mean gland areas of male and female cases for each cyst classification. Pineal glands with cysts of the fourth class were considerably larger than the glands in the other three categories (*p* < 0.0001, Mann-Whitney *U* test; appendix F).

**Table 3:** Classification of the cystic pineal gland according to the classification system shown in Table 1 with size suffix (a ≤5 mm, b 6–9 mm, c ≥10 mm) on a per-scan basis

Type	Suffix			Total (100 %)
	a	b	c	
1	103 (88.0 %)	12 (10.3 %)	2 (1.7 %)	117
2	23 (63.9 %)	13 (36.1 %)	0 (0.0 %)	36
3	29 (48.3 %)	31 (51.7 %)	0 (0.0 %)	60
4	11 (25.0 %)	22 (50.0 %)	11 (25.0 %)	44
Total	166 (64.6 %)	78 (30.4 %)	13 (5.1 %)	257

### Follow-up

**Fig. 5:**
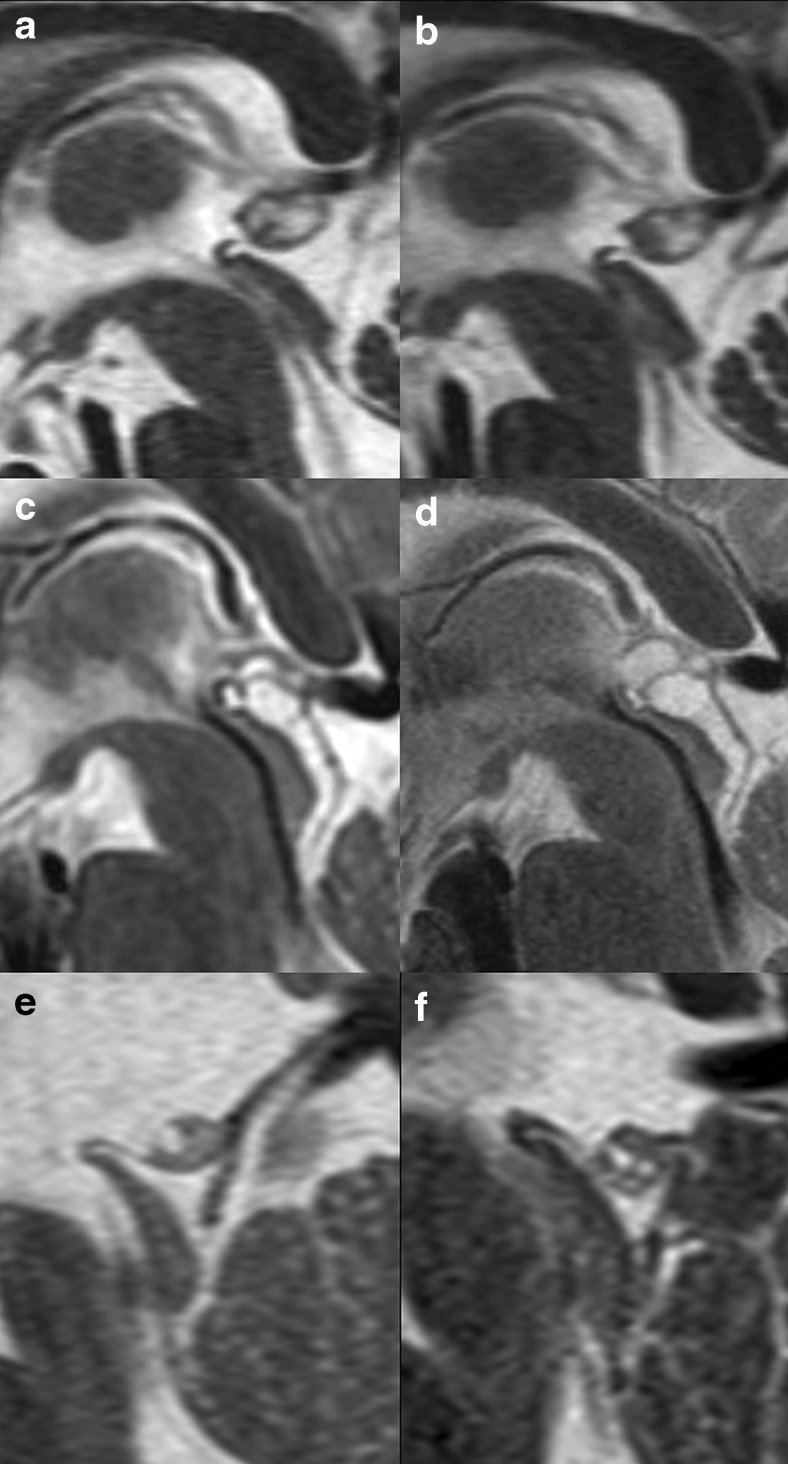
Follow-up of three patients with cystic pineal glands. **a** Polycystic pineal gland with decreasing cystic part especially in the anterior part of the cystic gland in the follow-up exam **b** after 11 months. **c** Polycystic pineal gland with an increase of the cystic part in the follow-up **d** after 28 months. **e** Initial exam of patient 1 with pineal gland type 1 (singular cyst) with development to a multicystic pineal gland in the follow-up exam after 47 months

Of the 25 children with available follow-up imaging (median 12 months, range 3 to 49 months, 11 males, 14 females), 48 % showed a singular pineal gland cyst and 52 % a multicystic pineal gland. Stable cystic findings were found in 48 %, cyst size increase in 36 % and decrease in 16 % (Fig. 5). Eighty-three percent (5/6) of the children with follow-up examinations performed within 6 months showed a stable finding (Table 4).In 2 children (17 %), the follow-up exam showed a development of the initial singular cyst to a multicystic pineal gland (Fig. 5). At both time points, all 25 children showed size parameters (width, height, area, and cyst size) that remained below the upper 99 % prediction bound.

**Table 4:** Pineal gland size changes over time

Time between examinations	Evaluation of the pineal gland size over time			Total
	Stable	Increase	Decrease	
<6 months	5 (83 %)	1 (17 %)	0 (0 %)	6 (24 %)
6–12 months	3 (50 %)	1 (17 %)	2 (33 %)	6 (24 %)
12–24 months	2 (40 %)	1 (20 %)	2(40 %)	5 (20 %)
24–36 months	1 (20 %)	4 (80 %)	0 (0 %)	5 (20 %)
36–48 months	0 (0 %)	2 (100 %)	0 (0 %)	2 (8 %)
48–60 months	1 (100 %)	0 (0 %)	0 (0 %)	1 (4 %)
Total	12 (48 %)	9 (36 %)	4 (16 %)	25 (100 %)

### Comparison with pineoblastoma

**Fig. 6:**
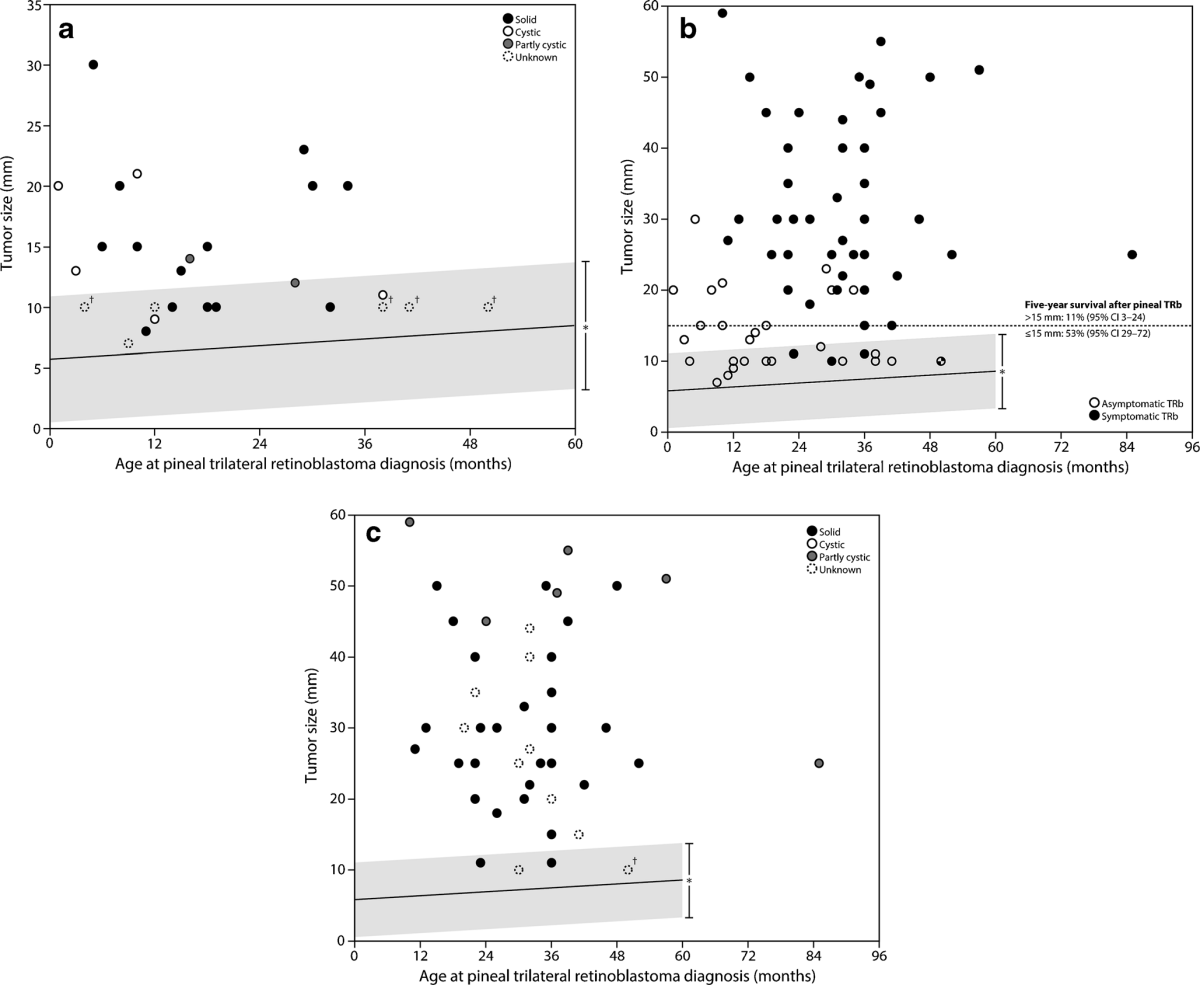
Cystic pineal gland width (mm) versus **a** the maximum diameter (mm) of only asymptomatic pineoblastomas, **b** of all pineoblastomas, and **c** of only symptomatic pineoblastomas. *TRb* = trilateral retinoblastoma. *99 % prediction intervals. †Of these pineoblastomas, the maximum diameter ranged from 5–15 mm

**Fig. 7:**
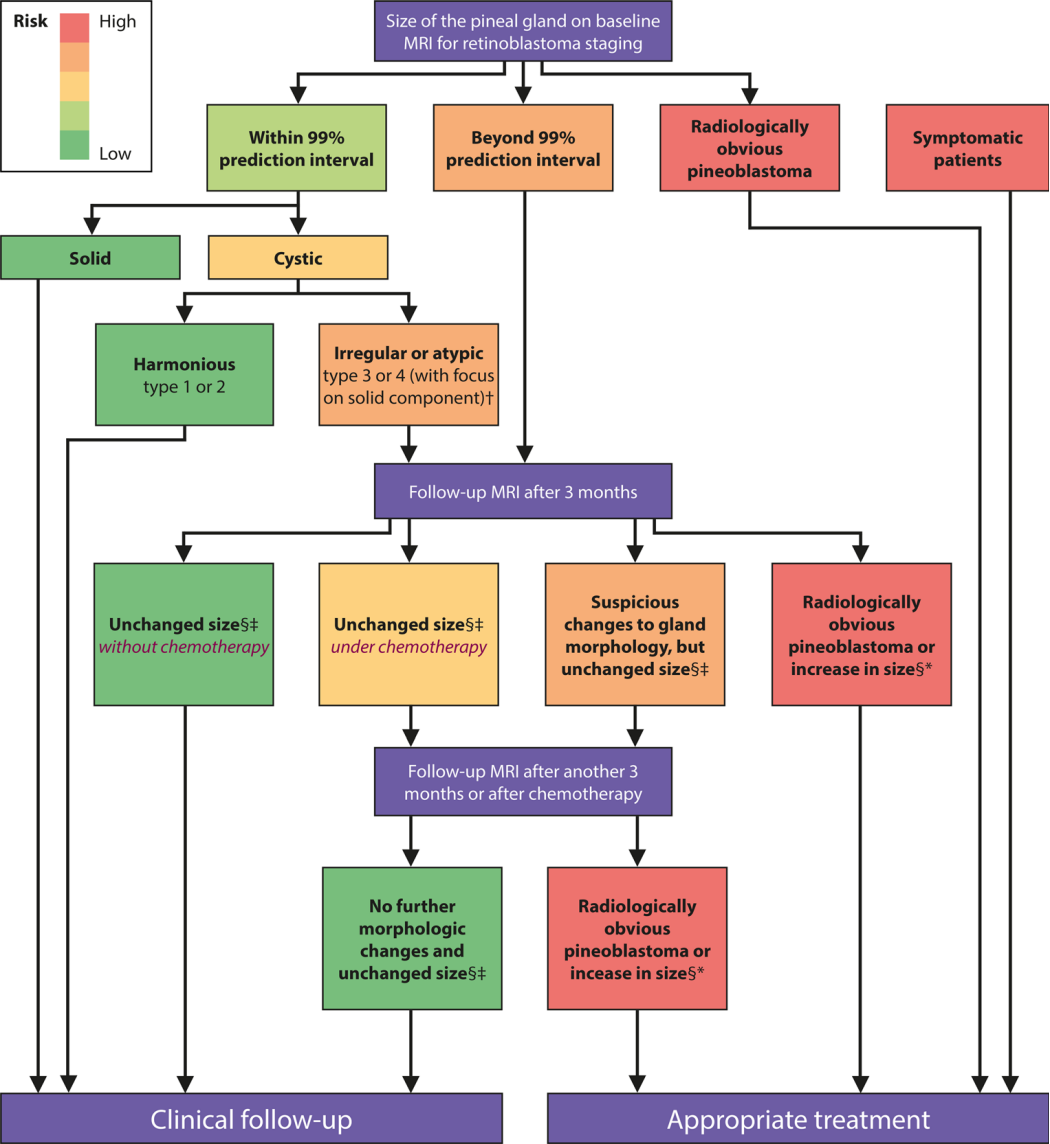
Consensus flowchart of the ERIC group for the evaluation, pineal gland follow-up, and necessity of treatment in children with retinoblastoma (sizes within the 99 % prediction intervals are considered normal for solid glands [part I] and type 1 and 2 cystic glands [this part]. †With a focus on the morphology: irregular or atypical aspect of the solid part in terms of shape, signal alteration, or irregular enhancement. §In case of a cystic gland, the focus of size change should be on the solid part. ‡Age-appropriate growth within 99 % prediction interval. *Not age appropriate/disproportionate

In Fig. 6, we compared the width of the normal cystic glands with the regression line with 99 % prediction interval with the maximum diameter of several pineoblastomas from which we were able to collect data. A considerable number of pineoblastomas overlapped in terms of size with the sizes of normal cystic pineal glands. Especially of interest are the cystic (*n* = 2), partly cystic (*n* = 1), and maybe those of unknown type (*n* = 6) in asymptomatic pineoblastomas (Fig. 6a). Three cystic and one partly cystic pineoblastoma had a larger maximum diameter than the 99 % upper prediction margin (Fig. 6a). In Fig. 7, we present our guidelines (flowchart) for the follow-up of the cystic and solid (see part I) pineal gland in children with retinoblastoma.

## Discussion

Cystic pineal glands are a frequent finding in children [7, 8] and are a challenge for radiologists and clinicians especially in the first years of life, because in this age group, rating of the size is difficult and imaging parameters can be very similar to cystic pineal pathologies [3, 13, 14]. We believe that knowledge of the normal size and morphology of the cystic pineal gland might be helpful for this differentiation. Until now, only a few studies exist evaluating the size of the pineal gland in childhood, mostly without differentiating between the solid and the cystic pineal gland and with low patient numbers between an age of 0 and 5 years [7, 8, 11, 15]. Additionally, different size parameters were used in the studies; therefore, the results are difficult to compare. We used measurements of height and width of the pineal gland and of the maximum cyst(s) size on sagittal high-resolution T2-weighted images and calculation of the planimetric area to provide a practical clinical approach. In our experience, it is more difficult to detect pineal cysts on postgadolinium T1-weighted images, because of the diffusion of contrast agent into the cysts. In accordance to the literature, age significantly predicted pineal size variables in our study [7, 8]. Interestingly, our data suggests a rapid increase of the pineal gland and the cyst size in the first year(s) of age and a decrease toward the fourth and fifth year of age. One possible explanation might be that melatonin (the principle secretory product of the pineal) reaches its highest levels at the age of 1–3 years and drops by 80 % by adolescence [16], which could be related to pineal gland size. Gender did not predict gland size in our study in accordance to prior studies [7, 8]. Altogether, our age-adapted normal values might serve as a reference standard for the size parameters of the pineal gland and the pineal cyst(s) in the first years of age.

Knowledge of the normal size of the pineal gland is important for the differentiation to cystic pineal pathologies. As the ERIC, we are especially interested in the differentiation of pineal cysts from cystic pineoblastoma. Normal values for pineal size of children without retinoblastoma are expected to be also applicable in children with retinoblastoma, because it has been shown that pineal gland size is comparable in retinoblastoma patients and age-matched controls [6]. Pineoblastoma typically develops within the first 5 years of life with an incidence of 3–4 % in children with hereditary retinoblastoma [2] and a much better survival has been shown for asymptomatic patients and patients with small tumors (≤15 mm) in a recent meta-analysis about trilateral retinoblastoma [1]. The comparison of our normal values of the cystic pineal gland size parameters to the available patients of this meta-analysis showed that some of the asymptomatic (partly) cystic pineoblastomas might have been classified as abnormal based on size alone. Therefore, we believe that our normal values may be helpful to detect and treat (partly) cystic pineoblastomas earlier with resulting better survival, as shown by the meta-analysis [1]. Nevertheless, other additional parameters such as evaluation of the solid part in terms of morphology (irregularity and changes over time), contrast enhancement, and MR signal intensity have to be evaluated further to identify those pineoblastomas with overlap in size. Our presented guidelines for follow-up might be helpful for a standardized evaluation of pineal glands in retinoblastoma.

It is the first classification system for the cystic pineal gland based on the number and the morphology of the pineal cysts. The incidence of multicystic pineal glands in children ranges from 3.6 to 74 % in the literature [9, 15], which might be a result of the different imaging parameters. In our patients, we found multicystic pineal glands in 53 %. All multicystic pineal glands with a maximum diameter of more than 1 cm of the cystic part interestingly showed a shift of the gland margin (type 4). The impact of this classification system for the cystic pineal gland for detecting pineoblastoma has to be evaluated in future studies, because the available data of the meta-analysis was insufficient to evaluate this aspect [1].

We were able to show that size and morphology of pineal gland cysts changed over time in our study as suggested before [7, 17]. Al-Holou et al. [12] showed that cysts are more likely to grow or change in younger compared to older children. Several mechanisms for pineal cyst size change have been proposed such as enlargement as a result of hormonal influence, due to hemorrhage or through remaining connection with the ventricular enlargement [8, 18]. Our findings of the pineal and cyst size development between the age of 0 and 5 years together with the results of the patients with follow-up especially support the first hypothesis, because cyst size decreased together with pineal gland size parameters after the age of 3, the time of the known decrease of melatonin [16]. These aspects have to be considered in the evaluation of the cystic pineal gland, because an increase of the cyst or pineal gland size, especially between the first and second year of life, should not be mistaken as pathologic enlargement.

Limitations of our study have to be acknowledged. We chose to focus only on the evaluation of size and morphology of the cystic pineal gland, because tumor size has been shown to be a prognostic factor for the outcome of children with pineal trilateral retinoblastoma, and abnormal growth of the pineal gland has been suggested to be the most alerting sign for pineoblastoma. We did not evaluate other parameters such as contrast enhancement or signal intensity; this was already done in prior studies [3–7, 15]. Additionally, due to the retrospective and multicenter character of this work, different MR parameters and scanners were used, which might have influenced our results.

In conclusion, we present age-adapted normal values for size and morphology of the cystic pineal gland in children aged 0–5 years without known pineal pathology or retinoblastoma that might be helpful in clinical routine and serve as comparison in future studies of (cystic) pineal pathologies. Analysis of pineal gland size is helpful in discriminating normal solid and cystic glands from pineoblastoma. We presented guidelines for the approach of a solid or cystic pineal gland in hereditary retinoblastoma patients.

## Electronic supplementary material

Below is the link to the electronic supplementary material.

ESM 1 (PDF 69 kb)

ESM 2 (PDF 49 kb)

ESM 3 (PDF 147 kb)

ESM 4 (PDF 399 kb)

ESM 5 (PDF 114 kb)
