# Supplementary material for: MRI-based assessment of the pineal gland in a large population of children aged 0–5 years and comparison with pineoblastoma: part II, the cystic gland
Source: Neuroradiology. 2016 Apr 29;58:713–21. doi: 10.1007/s00234-016-1683-0 (PMC4958131; doi:10.1007/s00234-016-1683-0)
Supplement: Supplementary file 1 — (PDF 69 kb) [file 234_2016_1683_MOESM1_ESM.pdf]

ONLINE APPEDICES

Appendix A. Two-way analysis of variance of gland sizes across age categories by gender.

**Width** ***(Lavene's test: p=0.19; adjusted R^2=0.22)***	**Sum of squares**	**Degrees of freedom**	**Mean square**	**F**	**P value**
Gender	3.70	1	3.70	1.03	0.31
Age	253.20	4	63.30	17.56	<0.0001
Gender*age	26.00	4	6.50	1.80	0.13
Within (error)	800.12	222	3.60		
Total	11929.39	232			
**Height** ***(Lavene's test: p=0.24; adjusted R^2=0.12)***	**Sum of squares**	**Degrees of freedom**	**Mean square**	**F**	**P value**
Gender	0.26	1	.26	.13	0.72
Age	66.25	4	16.56	8.60	<0.0001
Gender*age	13.96	4	3.49	1.81	0.13
Within (error)	427.39	222	1.93		
Total	5174.51	232			
**Area†** ***(Lavene's test: p=0.40; adjusted R^2=0.25)***	**Sum of squares**	**Degrees of freedom**	**Mean square**	**F**	**P value**
Gender	0.06	1	0.06	0.23	0.63
Age	20.21	4	5.05	20.06	<0.0001
Gender*age	1.66	4	0.41	1.64	0.16
Within (error)	55.91	222	0.25		
Total	2287.71	232			
**Cyst size‡** ***(Lavene's test: p=0.38; adjusted R^2=0.13)***	**Sum of squares**	**Degrees of freedom**	**Mean square**	**F**	**P value**
Gender	4.60	1	4.60	.732	0.39
Age	206.93	4	51.73	8.231	<0.0001
Gender*age	80.51	4	20.13	3.202	0.014
Within (error)	1395.25	222	6.28		
Total	6613.07	232			

†Log transformed to meet the homoscedasticity assumption. ‡Maximum diameter of the cyst(s) within the pineal gland.
