# Supplementary material for: MRI-based assessment of the pineal gland in a large population of children aged 0–5 years and comparison with pineoblastoma: part II, the cystic gland
Source: Neuroradiology. 2016 Apr 29;58:713–21. doi: 10.1007/s00234-016-1683-0 (PMC4958131; doi:10.1007/s00234-016-1683-0)
Supplement: Supplementary file 2 — (PDF 49 kb) [file 234_2016_1683_MOESM2_ESM.pdf]

Appendix B. Post hoc tests of the difference between age categories: Tukey's honestly significant difference test

**Age categories (months)**	**Age categories (months)**	**Cyst size (p value)**	**Width (p value)**	**Height (p value)**	**Area (p value)**
[0,12)	[12,24)	0.0002	<0.0001	0.0015	<0.0001
	[24,36)	0.0005	<0.0001	<0.0001	<0.0001
	[36,48)	0.0017	<0.0001	0.0059	<0.0001
	[48,60]	0.11	<0.0001	0.036	<0.0001
[12,24)	[0,12)	0.0002	<0.0001	0.0015	<0.0001
	[24,36)	0.98	0.46	0.95	0.79
	[36,48)	1.00	0.84	1.00	0.95
	[48,60]	0.54	0.99	0.94	1.00
[24,36)	[0,12)	0.0005	<0.0001	<0.0001	<0.0001
	[12,24)	0.98	0.46	0.95	0.79
	[36,48)	0.96	1.00	0.99	1.00
	[48,60]	0.80	0.76	0.54	0.70
[36,48)	[0,12)	0.0017	<0.0001	0.0059	<0.0001
	[12,24)	1.00	0.84	1.00	0.95
	[24,36)	0.96	1.00	0.99	1.00
	[48,60]	0.56	0.97	0.91	0.91
[48,60]	[0,12)	0.11	<0.0001	0.036	<0.0001
	[12,24)	0.54	0.99	0.94	1.00
	[24,36)	0.80	0.76	0.54	0.70
	[36,48)	0.56	0.97	0.91	0.91
