# Supplementary figures and images for: MRI-based assessment of the pineal gland in a large population of children aged 0–5 years and comparison with pineoblastoma: part II, the cystic gland
Source: Neuroradiology. 2016 Apr 29;58:713–21. doi: 10.1007/s00234-016-1683-0 (PMC4958131; doi:10.1007/s00234-016-1683-0)

**Appendix C.** Mean size of the pineal size variables for each age category.

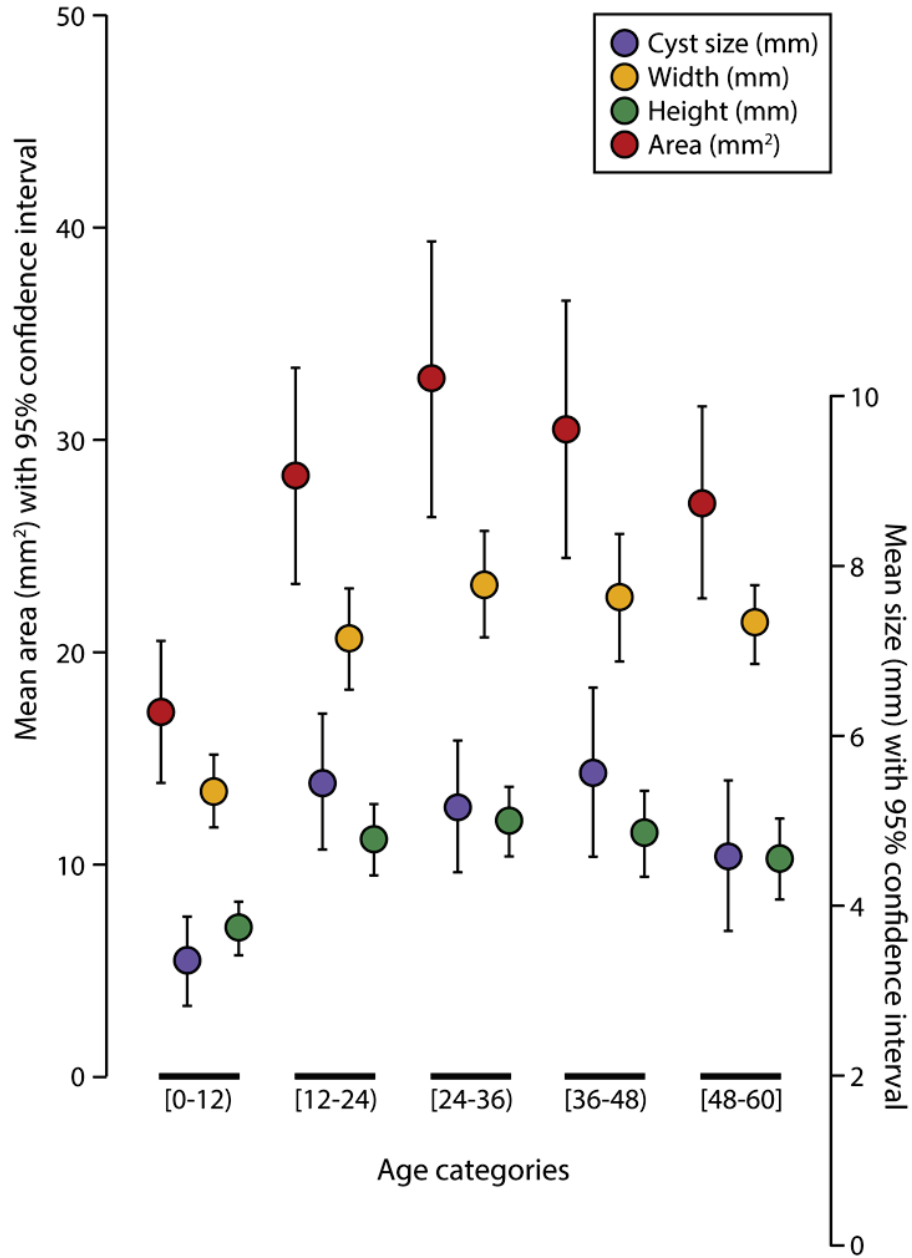

Supplement: Supplementary file 3 — (PDF 147 kb) [file 234_2016_1683_MOESM3_ESM.pdf]
