# Supplementary material for: MRI-based assessment of the pineal gland in a large population of children aged 0–5 years and comparison with pineoblastoma: part II, the cystic gland
Source: Neuroradiology. 2016 Apr 29;58:713–21. doi: 10.1007/s00234-016-1683-0 (PMC4958131; doi:10.1007/s00234-016-1683-0)
Supplement: Supplementary file 4 — (PDF 399 kb) [file 234_2016_1683_MOESM4_ESM.pdf]

Appendix D. Quadratic regression line with 99% prediction intervals of width (A), height (B), area (C) and cyst size (D).

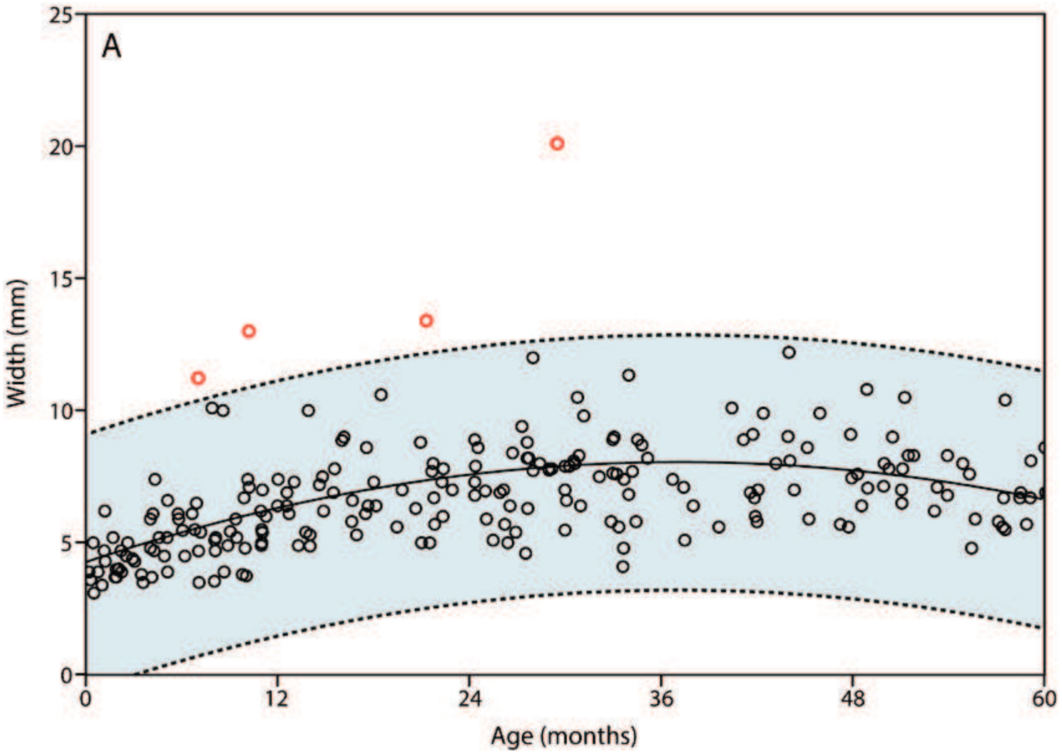

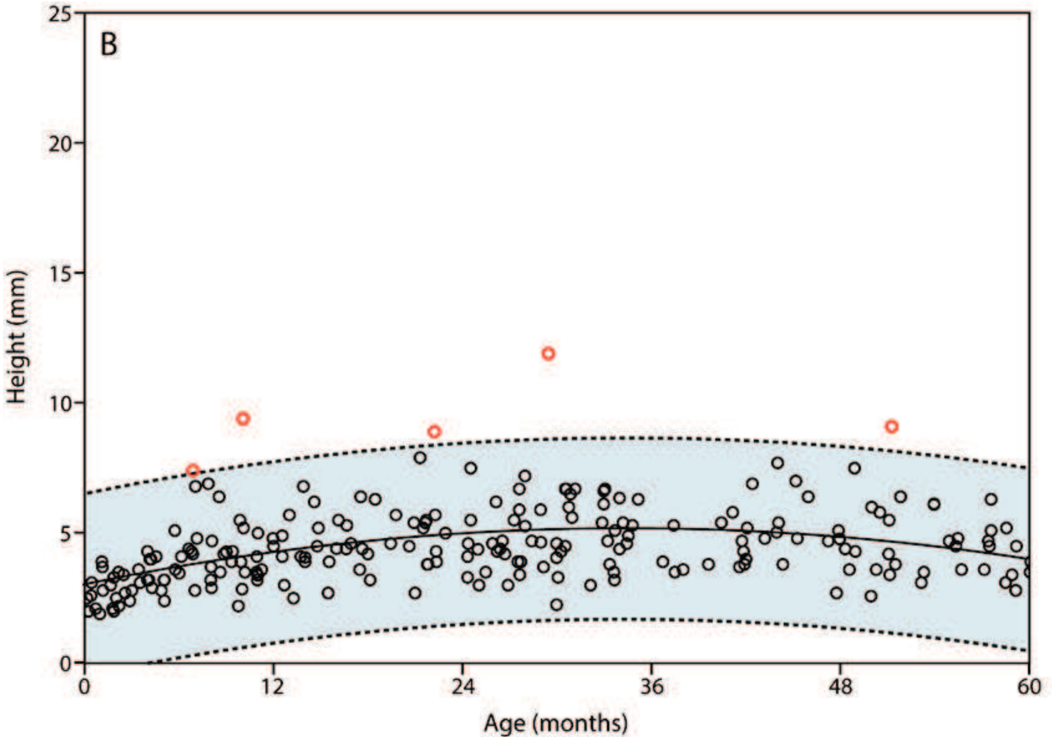

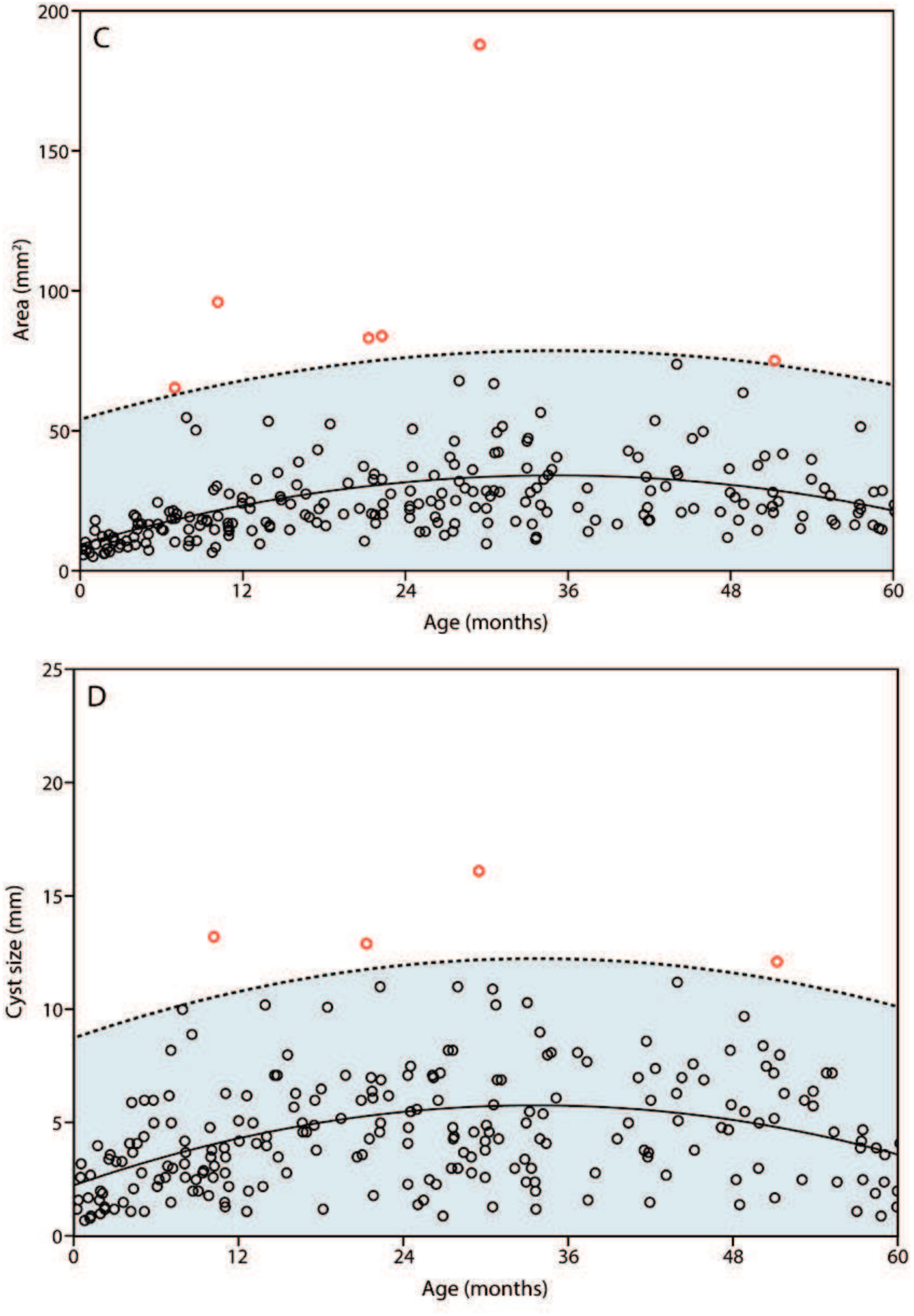
C
Area (mm²)
200
150
100
50
0
0
12
24
36
48
60
Age (months)
D
Cyst size (mm)
25
20
15
10
5
0
0
12
24
36
48
60
Age (months)
