# Supplementary material for: MRI-based assessment of the pineal gland in a large population of children aged 0–5 years and comparison with pineoblastoma: part II, the cystic gland
Source: Neuroradiology. 2016 Apr 29;58:713–21. doi: 10.1007/s00234-016-1683-0 (PMC4958131; doi:10.1007/s00234-016-1683-0)

Appendix E. Results of quadratic regression analysis: cystic pineal gland size versus age.

Relationship	Mean intercept (mm)	Upper bound (mm)*	Coefficient (mm/month)	P value	Coefficient (mm/month^2)	P value	Adjusted R^2
Cyst size‡ vs. age	2.3	8.8	0.209	<0.0001	-0.00311	<0.0001	0.15
Width vs. age	4.3	9.2	0.202	<0.0001	-0.00271	<0.0001	0.26
Height vs. age	3.0	6.6	0.126	<0.0001	-0.00182	<0.0001	0.18
**Relationship**	**Mean intercept (mm^2)**	**Upper bound (mm^2)***	**Function (mm^2/month)**	**p value**	**Function (mm^2/month^2)**	**p value**	**Adjusted R^2**
Area vs. age	9.3	54.7	1.410	<0.0001	-0.02008	<0.0001	0.15

*The upper 99% prediction bound has approximately the same function as the regression line; therefore the slope of the linear regression line can be used. ‡Maximum diameter of the cyst(s) within the pineal gland.

Appendix F. Mean gland areas of male and female cases for each cyst classification.

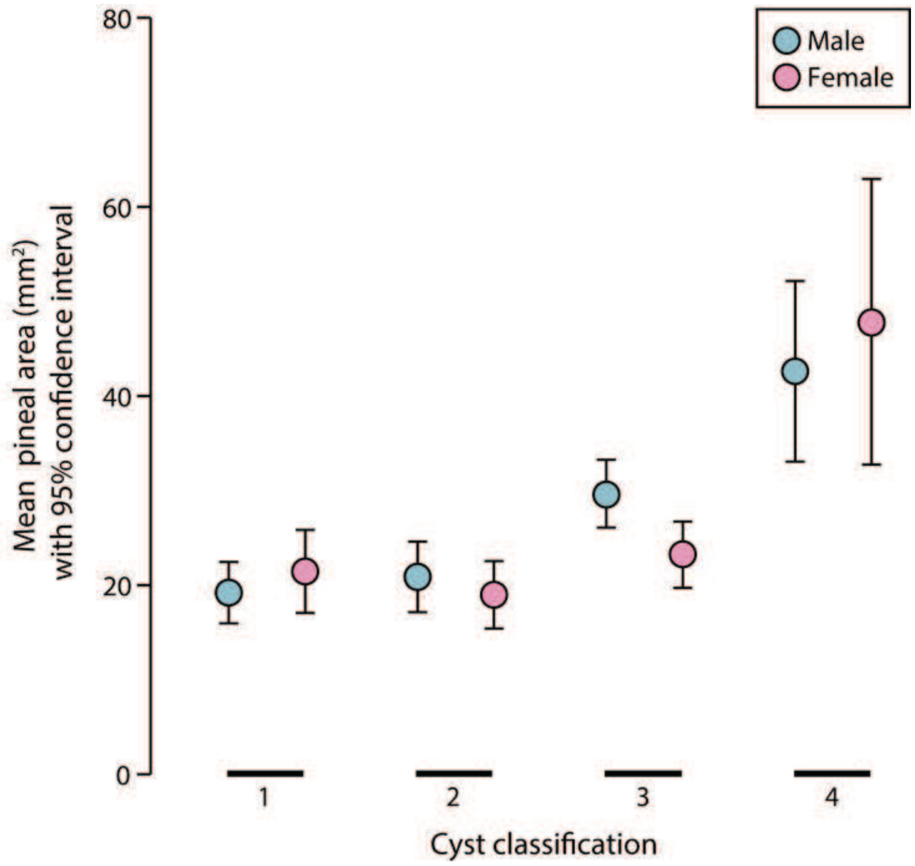

Supplement: Supplementary file 5 — (PDF 114 kb) [file 234_2016_1683_MOESM5_ESM.pdf]
